# Evidence for the biopsychosocial model of suicide: a review of whole person modeling studies using machine learning

**DOI:** 10.3389/fpsyt.2023.1294666

**Published:** 2024-01-11

**Authors:** Earvin S. Tio, Melissa C. Misztal, Daniel Felsky

**Affiliations:** ^1^Krembil Centre for Neuroinformatics, Centre for Addiction and Mental Health, Toronto, ON, Canada; ^2^Institute of Medical Science, Temerty Faculty of Medicine, University of Toronto, Toronto, ON, Canada; ^3^Biostatistics Division, Dalla Lana School of Public Health, University of Toronto, Toronto, ON, Canada; ^4^Department of Psychiatry, Temerty Faculty of Medicine, University of Toronto, Toronto, ON, Canada

**Keywords:** suicide, suicidal ideation, machine learning, whole person, review

## Abstract

**Background:**

Traditional approaches to modeling suicide-related thoughts and behaviors focus on few data types from often-siloed disciplines. While psychosocial aspects of risk for these phenotypes are frequently studied, there is a lack of research assessing their impact in the context of biological factors, which are important in determining an individual’s fulsome risk profile. To directly test this biopsychosocial model of suicide and identify the relative importance of predictive measures when considered together, a transdisciplinary, multivariate approach is needed. Here, we systematically review the emerging literature on large-scale studies using machine learning to integrate measures of psychological, social, and biological factors simultaneously in the study of suicide.

**Methods:**

We conducted a systematic review of studies that used machine learning to model suicide-related outcomes in human populations including at least one predictor from each of biological, psychological, and sociological data domains. Electronic databases MEDLINE, EMBASE, PsychINFO, PubMed, and Web of Science were searched for reports published between August 2013 and August 30, 2023. We evaluated populations studied, features emerging most consistently as risk or resilience factors, methods used, and strength of evidence for or against the biopsychosocial model of suicide.

**Results:**

Out of 518 full-text articles screened, we identified a total of 20 studies meeting our inclusion criteria, including eight studies conducted in general population samples and 12 in clinical populations. Common important features identified included depressive and anxious symptoms, comorbid psychiatric disorders, social behaviors, lifestyle factors such as exercise, alcohol intake, smoking exposure, and marital and vocational status, and biological factors such as hypothalamic-pituitary-thyroid axis activity markers, sleep-related measures, and selected genetic markers. A minority of studies conducted iterative modeling testing each data type for contribution to model performance, instead of reporting basic measures of relative feature importance.

**Conclusion:**

Studies combining biopsychosocial measures to predict suicide-related phenotypes are beginning to proliferate. This literature provides some early empirical evidence for the biopsychosocial model of suicide, though it is marred by harmonization challenges. For future studies, more specific definitions of suicide-related outcomes, inclusion of a greater breadth of biological data, and more diversity in study populations will be needed.

## 1 Introduction

Suicide is a global public health crisis, with over 700,000 estimated deaths attributed to suicide annually (1). Despite decades of research into suicidal thoughts and behaviors, predicting risk for suicide remains a challenge (2, 3). Suicidal thoughts and behaviors can be classified into suicidal ideation (SI), suicide attempt (SA), and completed suicide, with nonsuicidal self-injury often considered a separate disorder with a potentially unique etiology (4). Commonly studied risk factors for suicidal thoughts and behaviors include previous self-injurious thoughts and actions (5), previous SA (6), and adverse life events (7). Previous attempts are also known risk factors for future attempts (6), alongside various social factors – such as childhood mistreatment (8) and job security (9) – and psychiatric comorbidities (10). In particular, personality, alcohol use, and anxiety disorders (10, 11) increase risk for SA, while depressive disorders are indicative of SI without associated plans or attempts (10). Social factors in particular – such as childhood maltreatment (8), hopelessness, and interpersonal and emotion dysregulation (12) – have also been shown to contribute to SI. Psychiatric comorbidities are also risk factors for completed suicides, with depression, substance use, and psychosis being most relevant (13). These risk factors are rooted in psychosocial domains, and preventative or interventional strategies based on these findings have found only limited success (14, 15). A critical, often missing consideration in observational studies, is that suicide is a complex trait, resulting from the complex interactions of both intrinsic biological and extrinsic environmental components (2, 16). Relative to social and psychological contributions, the intrinsic biological contributions, remain poorly understood.

Twin studies have shed some light on the genetic components of suicide-related phenotypes, with heritability estimates ranging from 17–55% (17, 18); heritability varies depending on the specific phenotype under study, with completed suicide on the lower end (17%) (19), followed by SA (30%) (20), SI (47%) (20), and serious attempt (55%) (21). Recent large-scale genome-wide analyses have identified two risk loci associated with SA (residing in the major histocompatibility complex region on chromosome 6 and an intergenic locus on chromosome 7) and estimate single-nucleotide polymorphism (SNP)-based heritability at 6.8% (22). Reviews of brain imaging studies using MRI to classify current or lifetime SI, SA, and suicide risk have identified changes in resting-state functional connectivity and differences in prefrontal-limbic grey matter volume (23). For attempts in particular, smaller volumes of the left and right thalamus and the right pallidum, and lower surface area of the left inferior parietal lobe have been observed (24). Putative blood-based biomarkers (25), particularly inflammatory markers such as C-reactive protein, interleukin 6, and tumor necrosis factor-alpha (26, 27), have also been identified, broadening the range of biomarkers that correlate with and predict suicide phenotypes. Yet, despite mounting evidence from biological, psychological, and social disciplines, models of suicide in human studies rarely adopt an integrative, transdisciplinary perspective. Instead, the majority of research has focused on only one or two types of risk factors, most often clinical or psychological (28). Conversely, recent advancements in our understanding of the biological underpinnings of suicide are usually marred by a lack of consideration of psychosocial context; these findings must be contextualized to the experiences of the individual to ultimately facilitate a precision approach to suicide prevention (29). Thus, assessment of risk in predictive modeling of suicide stands to benefit from including transdisciplinary information, integrating across domains of measurement simultaneously.

Such study designs – falling under the umbrella of Whole Person Modeling (30) – are enabled by large-scale biobank and clinical cohort initiatives collecting data from electronic health records (EHR), multi-modal biosamples, and detailed sociodemographic surveys, neuropsychological assessments, and lifestyle questionnaires (31, 32). The size and breadth of the resulting datasets have also permitted the application of advanced, multivariate, machine learning (ML) approaches to the modeling of suicide-related outcomes (33). For example, Walsh et al. (34, 35) trained random forest models for prediction of SA using data drawn from the BioVU Synthetic Derivative, a de-identified data repository of clinical EHR data at Vanderbilt University Medical Center (36), using a longitudinal approach. The authors focused on scalable and temporally-variant predictors and chose to exclude biological features, such as vital signs, which often reflect more immediate, trait-like responses. Similarly, Kessler et al. (37) trained regression trees on data drawn from the Historical Administrative Data System (HADS) of the Army Study to Assess Risk and Resilience in Servicemembers (Army STARRS) (38). The authors referred to published literature to identify five main classes of predictors: (1) sociodemographics, (2) history of prior suicidal behaviors, (3) quality of care, (4) time since hospital discharge, and (5) other psychopathological risk factors. They also chose to include military- and violence-specific measures due to the population under study. While these and other ML-based studies often demonstrate high predictive performance (with accuracies approaching or beyond 80%) they lack integration of biological information, therefore leaving important chips on the table when it comes to informing treatable mechanisms, developing objective biomarkers of risk and prognosis, and ultimately achieving clinically reliable levels of model performance.

Modeling of suicide-related phenotypes using ML and a Whole Person approach may provide insights into the complex landscape of risk factors arising from interactions of genome, phenome, and exposome (30). Beyond what is possible with simpler linear, hypothesis-testing methods that dominate the extant literature, such an approach is necessary to understand the influences of biopsychosocial risk factors when considered in consort. Recent reviews have shown that ML models are capable of identifying scientific insights into suicidology and outperform theory-driven models in predicting suicide-related outcomes (39, 40). Therefore, this review aims to summarize the burgeoning literature of human suicide-related modeling studies that both (a) use ML approaches capable of handling multiple data types, and (b) adopt a Whole Person, or transdisciplinary, approach considering biopsychosocial measures together in their feature space. The principal goals of this review are to understand which populations are most studied, which features emerge most consistently as predictors of suicide-related phenotypes, and what evidence can be gleaned from this work in favor of or against the biopsychosocial model of suicide.

## 2 Materials and methods

### 2.1 Conceptual definitions

To identify published papers meeting our criteria of using biopsychosocial data types to model suicide-related phenotypes, we first outline our key considerations in defining biopsychosocial domains. In accordance with the tenets of Whole Person Modeling (30), we considered biological features to be separate from clinical diagnostic categories or prescribed medications. For example, features such as comorbid somatic diseases (e.g., cancer or diabetes) are not themselves considered biological risk factors, despite their biological basis. Similarly, medication burden and treatment history (e.g., having undergone surgery) are clinically-defined consequences of underlying etiological processes, signs, and symptoms, and not biomarkers in and of themselves. Some examples of biological risk factors are genetic factors, such as pathological variants identified through genome-wide association studies (GWAS); polygenic risk scores that aggregate GWAS findings across the whole genome; transcriptomic or proteomic measures, such as tissue-specific differentially expressed genes; neuroimaging-derived features such as cortical thickness or functional connectivity (i.e., from MRI); or fluid biomarkers, such as results from a complete blood count or levels of circulating inflammatory molecules.

Within the psychological domain, we considered comorbid psychiatric diagnoses as component risk factors for suicide and suicidal thoughts and behaviors. This is primarily due to the lack of consensus from the field regarding whether suicide is etiologically distinct from other psychiatric disorders, or rather a manifestation of a shared transdiagnostic mechanism, akin to the “p” factor for psychopathology (41, 42). Ideally, psychological risk factors are derived from observed psychological behaviors and reports, such as depressive or anxious symptom presentation indexed with validated tools like the Hamilton Rating Scales for depression and anxiety (HAM-D and HAM-A, respectively) (43, 44).

Finally, risk factors in the sociological domain are heterogeneous, ranging from socioeconomic status and income levels to features of familial and personal relationships. These are often collectively referred to as social determinants of health (45) and are critical in understanding mental health (46), especially in a public context (47). However, due to an unfortunate historical lack of emphasis on this domain in suicide research (28), we relaxed our criteria for sociological risk factors when considering inclusion of a report for this review (i.e., considering education level and marital status as sufficient sociological variables in some cases).

### 2.2 Literature search strategy

We performed a systematic literature review in the electronic databases PubMed, MEDLINE, EMBASE, PsychINFO, and Web of Science for reports published up to August 30, 2023 following the Preferred Reporting Items for Systematic reviews and Meta-Analysis (PRISMA) (48) guidelines. Results were restricted to publications in English and published since August, 2013 (i.e., within the past ten years). Comprehensive search strategies for each electronic database can be found in Supplementary Table S1. Briefly, our search strategy included Medical Subject Headings (MeSH) and keywords related to three concepts: suicide (using a wild card to match for suicidality, suicidal ideation, and suicide attempt, among other suicide-related terms), artificial intelligence (or machine learning or deep learning), and prediction or classification (again using wild cards to match for predictors or classifiers as we are interested in the predictive feature space of the models). EST first screened the articles by title and abstract, followed by independent review of the selected full text by two authors (EST and MCM). Differences in full-text review between the authors are captured in the risk of bias assessment (Supplementary Tables S2, S3). This did not impact the final selection of included studies.

### 2.3 Eligibility criteria

Studies were included if they met the following inclusion criteria: (1) the primary modeled outcome was clearly defined as suicidal ideation, suicide attempt or completion, or risk, measured on a validated scale; (2) the input feature space included at least one predictor derived from each of the following three domains: biological, psychological, and sociological; and (3) the study used an ML-based approach to combine these factors together, rather than evaluating them independently. Studies on non-human subjects, review papers, case reports or qualitative studies, dissertations, opinion pieces, comments or responses, letters or editorials, and conference abstracts or posters were excluded.

### 2.4 Data extraction

The following data from eligible studies were manually extracted: bibliographic information (i.e., title, names of authors, year of publication, and journal), suicide-related outcome and how it was ascertained, sample characteristics, details on the ML method used, and model features (i.e., predictors) categorized by domain.

### 2.5 Risk of bias assessment

Quality assessment of all included studies was performed using the Risk of Bias Instrument for Cross-Sectional Surveys of Attitudes and Practices developed by the CLARITY Group of McMaster University (49). The assessment tool includes five questions, each on a four-point Likert scale ranging from low risk of bias to high risk of bias. Two authors (EST and MCM) independently assessed risk of bias in the included studies, modified for some studies where assessment of survey validity is not applicable (i.e., questions 4 and 5 were left blank). Interrater reliability was calculated with Cohen’s kappa statistic (50) by coding the Likert-scale responses on a 1-to-4 point scale and dichotomizing to low- or high-risk of bias.

## 3 Results

**Figure 1:**
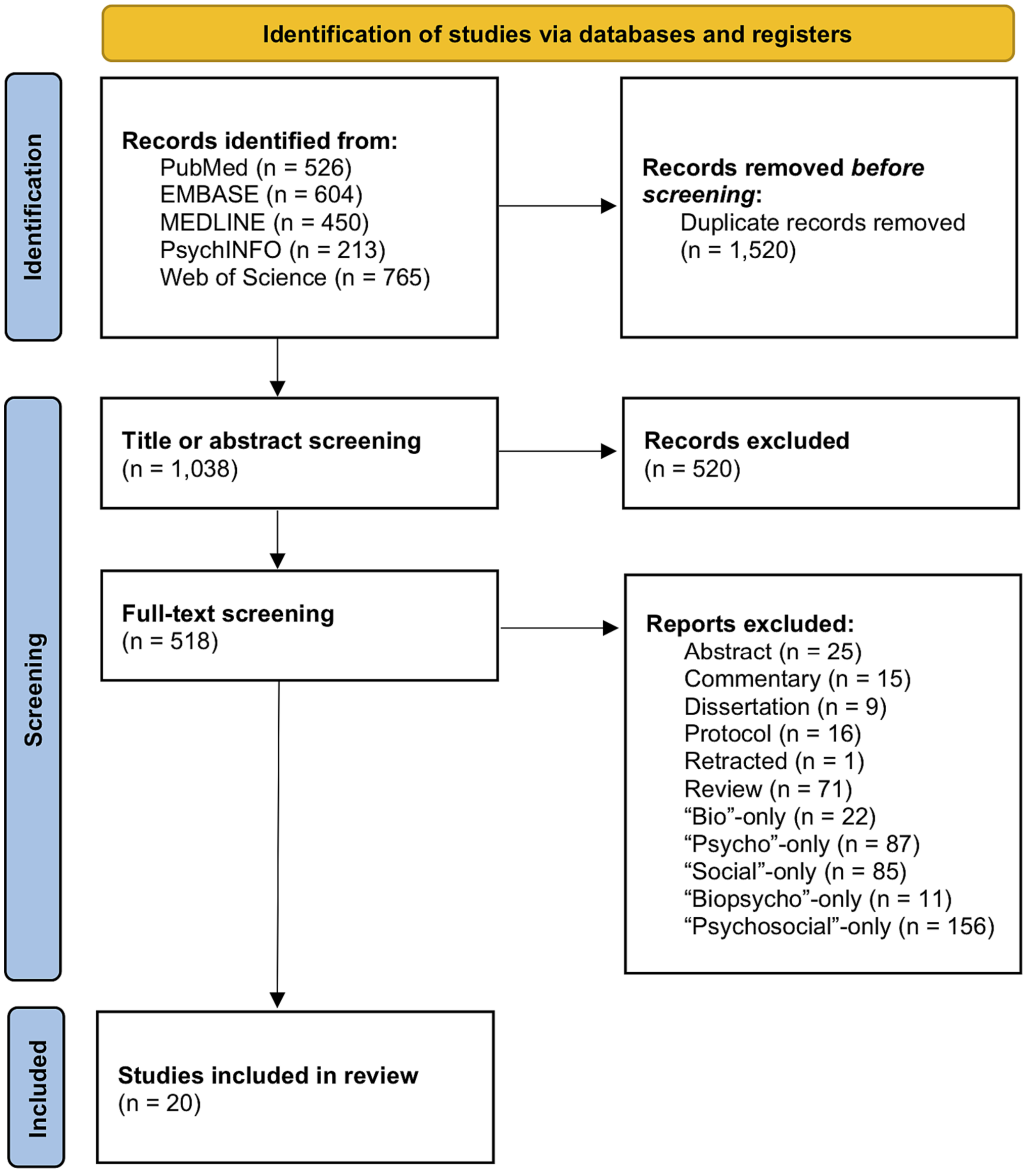
PRISMA flow chart of identified records and the various screening stages for eventual selection of the included studies.

A total of 1,038 unique reports were identified after the removal of duplicates. After title and abstract screening, 518 full-text articles were assessed for eligibility, and a final set of 20 studies were included in this systematic review. The PRISMA flow chart illustrating inclusion and exclusion of reports at various stages of screening can be found in Figure 1. Table 1 presents the characteristics of the final set of studies. The features presented in Table 1 are not exhaustive – some of the included studies investigate hundreds of potential predictive features. The listed features are meant to highlight top statistical predictors found in each study to emphasize the Whole Person, biopsychosocial approach taken. Model performance metrics and information regarding feature importance selection can be found in Supplementary Table S4.

**Table 1:** Whole person studies of suicide-related outcomes using machine learning with representative features.

Authors	Title	Journal	Year	Study design	Population	Outcome	Machine learning	Biological	Psychological	Sociological
Allesøe et al. (53)	Deep Learning for Cross-Diagnostic Prediction of Mental Disorder Diagnosis and Prognosis Using Danish Nationwide Register and Genetic Data	JAMA Psychiatry	2023	Retrospective cross-sectional population-based case-cohort study	Danish general population	Suicide attempt and completed suicide	Feed-forward neural network	Genotype data, polygenic risk score, and medical birth registry data	Comorbid psychiatric diagnoses with age of onset	Family diagnosis history
Barak-Corren et al. (65)	Improving risk prediction for target subpopulations: Predicting suicidal behaviors among multiple sclerosis patients	PLOS One	2023	Retrospective cross-sectional cohort study design	Patients with Multiple Sclerosis (ICD-9 of 340)	Suicide attempt (ICD-9 codes: E95*, 965.*, 967.*, 969.*, 881.*) and completed suicide (ICD-9: E95* or ICD-10 ×60-X84, Y87.0)	Naive Bayesian classifier	Laboratory tests	Psychiatric diagnoses	Marital status
Edgcomb et al. (66)	Assessing Detection of Children With Suicide-Related Emergencies: Evaluation and Development of Computable Phenotyping Approaches	JMIR Mental Health	2023	Observational cross-sectional study	Children aged 10–17 years who presented in an American ED	Presence or absence of SITB at time of ED visit from clinician chart reviews	LASSO regression and random forest	Laboratory tests	Mental health-related diagnostic or billing codes, chief complaint	Area deprivation index
Lyall et al. (59)	Subjective and objective sleep and circadian parameters as predictors of depression-related outcomes: A machine learning approach in UK Biobank	Journal of Affective Disorders	2023	Retrospective cross-sectional population-based cohort study	British general population with reported depression diagnosis	Self-reported suicidal thoughts and behaviors in depressed individuals	LASSO, ridge, and elastic net penalized regression; logistic regression	Objective actigraphy-based sleep/rest-activity	Subjective sleep/ chronotype characteristics	Townsend deprivation score
Roza et al. (60)	Suicide risk classification with machine learning techniques in a large Brazilian community sample	Psychiatry Research	2023	Retrospective cross-sectional population-based cohort study	Public institution employees from six major Brazilian cities with common mental disorders	Suicide risk measured by three questions from the Clinical Interview Schedule-Revised, adapted Brazilian Portuguese version	Elastic net regularization, random forest, Naive Bayes, and ensemble	Biomarkers originally focused on cardiovascular disease (i.e., LDL or carotid artery intima-media thickness)	Emotional difficulties, mental health variables	Marital status, socioeconomic status, social capital
Wang et al. (54)	Prediction of Suicidal Behaviors in the Middle-aged Population: Machine Learning Analyses of UK Biobank	JMIR Public Health and Surveillance	2023	Retrospective longitudinal case–control study design	British general population	Suicide attempt (ICD 10: X60-84 and Y10-34; ICD-9: E950-958) and completed suicide (ICD-10: X60-84 and Y10-34; ICD-9: E950-958)	Light gradient-boosting machine with balanced bagging	Genetic susceptibility via polygenic risk score for suicidality	Psychiatric illness history and subjective mental health	Familial characteristics, derived dietary pattern
Yang et al. (61)	Establishment of a risk prediction model for suicide attempts in first-episode and drug naïve patients with major depressive disorder	Asian Journal of Psychiatry	2023	Retrospective cross-sectional study	Patients of a psychiatric outpatient department of a general hospital in China	Suicide attempt as indicated by clinical interviews	LASSO regression	Lipid assays, hypothalamic–pituitary-thyroid axis activity, and plasma glucose	Depressive symptoms, anxiety symptoms, and psychotic symptoms measured by various validated scales	Educational level and marital status
Balbuena et al. (67)	Identifying long-term and imminent suicide predictors in a general population and a clinical sample with machine learning	BMC Psychiatry	2022	Retrospective longitudinal study	Population-based cohort of Norway and a cohort of people who ever visited a Saskatoon hospital for mental health or substance-related reason	Suicide as defined by ICD-10 codes X60-X84 or Y87.0	Cox regression models and random survival forests	Triglycerides, HDL-cholesterol, glucose, and total cholesterol	Mood symptoms measured by the Hopkins Symptom Checklist	Years of education and relative social deprivation
Campos et al. (62)	Clinical, demographic, and genetic risk factors of treatment-attributed suicidality in >10,000 Australian adults taking antidepressants	American Journal of Medical Genetics	2022	Retrospective cross-sectional cohort study	People with depression living across Australia	Treatment-associated suicidal ideation as measured by the Antidepressant Efficacy and Side Effects Questionnaire	Naive Bayes, decision tree, adaptive boosting, random forests, and logistic regression	Genome-wide associations	Depressive symptoms, antidepressant use	Marital status
Grendas et al. (68)	Comparison of traditional model-based statistical methods with machine learning for the prediction of suicide behavior	Journal of Psychiatric Research	2022	Retrospective longitudinal study	Patients admitted to an emergency department in Argentina for active suicidal ideation or a recent suicide attempt	Subsequent suicide or a suicide reattempt within a follow-up period	Cox regression models and random survival forests	Genotype data	Psychiatric illness and psychiatry treatment history, lifetime and recent suicidal thoughts and behaviors, impulsivity, hostility, and hopelessness measured by various validated scales	Recent stressors measured by the Brugha Stressful Life Events Scale and psychosocial functioning measured by the Social Adaptation Self-evaluation Scale
Joo et al. (51)	Association of Genome-Wide Polygenic Scores for Multiple Psychiatric and Common Traits in Preadolescent Youths at Risk of Suicide	JAMA Network Open	2022	Retrospective cross-sectional cohort study	Children aged 9–10 years recruited across 21 sites in the U.S.	Suicidal ideation (active, passive, and overall) and suicide attempt measured by the Kiddie Schedule for Affective Disorders and Schizophrenia for DSM-5	Multivariate logistic regression, random forest, and elastic net regression	Polygenic risk scores	Psychological observations measured by the Child Behavior Checklist and early life stress scores	Family income, parental marital status, familial characteristics measured by the Family Environment Scale
Lozupone et al. (57)	Apolipoprotein E genotype, inflammatory biomarkers, and non-psychiatric multimorbidity contribute to the suicidal ideation phenotype in older age. The Salus in Apulia Study	Journal of Affective Disorders	2022	Retrospective cross-sectional cohort study	Population-based elderly (65+) cohort of community-dwellers in Castellana Grotte, in the Puglia region of South East Italy	Suicidal ideation measured by the Columbia-suicide severity rating scale (C-SSRS)	Random forest	Metabolic and immunoassay data from fasted blood samples, genotype data	Standardized neuropsycho-logical tests and the Mini Mental State Examination	Social deprivation measured by the Deprivation in Primary Care Questionnaire
Tate et al. (55)	A genetically informed prediction model for suicidal and aggressive behavior in teens	Translational Psychiatry	2022	Retrospective cross-sectional population-based cohort study	Population-based twin cohorts (Sweden and Netherlands)	Suicidal behaviors indicated by specific questions from the Life History of Aggression Checklist (Sweden) or the Young Adult Self Report and Adult Self Report of the Achenbach System of Empirically Based Assessment (Netherlands) at age 18	Gradient boosted machine, random forest, elastic net, and a neural network	Polygenic risk scores	Psychiatric symptoms and substance use	Parent and child relationship characteristics
van Velzen et al. (52)	Classification of suicidal thoughts and behavior in children: results from penalized logistic regression analyses in the Adolescent Brain Cognitive Development study	The British Journal of Psychiatry	2022	Retrospective cross-sectional cohort study	Children aged 9–11 years recruited across 21 sites in the U.S.	Lifetime parent- or child-reported suicidal thoughts or behavior recorded by the Kiddie Schedule for Affective Disorders and Schizophrenia for DSM-5	Binomial penalized logistic regression	Genetic and neuroimaging (task MRI) data	Dimensional mania symptoms as assessed by the parent-reported Mania Scale from the Parent General behavior Inventory, current and past psychiatric diagnoses, and cognitive scores	Parent and child relationship characteristics, prosocial and bullying behaviors, school environment, and neighborhood safety
Cho et al. (58)	Development of a Suicide Prediction Model for the Elderly Using Health Screening Data	International Journal of Environmental Research and Public Health	2021	Retrospective cross-sectional cohort study	Elderly (65+) users of the National Health Insurance Sharing Service of South Korea	Suicide as defined by ICD-10 codes X60–X84 or Y10–Y34	Random forest	Fasting plasma glucose, total cholesterol, triglyceride, creatinine, hemoglobin, aspartate transaminase, and gamma-glutamyl transpeptidase	Psychiatric diagnoses and psychiatric drug use	Disability, medical benefits, ranking of insurance premium payment
Li et al. (63)	Identifying clinical risk factors correlate with suicide attempts in patients with first episode major depressive disorder	Journal of Affective Disorders	2021	Retrospective cross-sectional study design	First-episode, untreated MDD patients recruited from the Department of Psychiatry, First Clinical Medical College of Shanxi Medical University in Taiyuan, China	Attempt (recent vs. long-dated) vs. non-attempters	Gradient boosted decision trees with extreme gradient boosting	Lipid assays, hypothalamic–pituitary-thyroid axis activity, and plasma glucose	Depressive symptoms, anxiety symptoms, severity of disease, and psychotic symptoms measured by various validated scales	Education level and marital status
Oppenheimer et al. (69)	Informing the study of suicidal thoughts and behaviors in distressed young adults: The use of a machine learning approach to identify neuroimaging, psychiatric, behavioral, and demographic correlates	Psychiatry Research: Neuroimaging	2021	Recruitment-based cross-sectional study design	Help-seeking (i.e., mental health counseling, psychiatric distress) young adults (18–25 years) recruited from the general public	Suicidal thoughts and behaviors measured by the 6-item suicidality subscale of the Mood Spectrum Self-Report questionnaire	LASSO regression	fMRI-derived measures	Anxiety symptoms, depressive symptoms, impulsivity, mania, and psychological distress measured by various validated scales	Education level
Cho et al. (56)	Prediction of suicide among 372,813 individuals under medical check-up	Journal of Psychiatric Research	2020	Retrospective cross-sectional cohort study	Users of the National Health Insurance Sharing Service of South Korea	Suicide as defined by ICD-10 codes X60–X84 or Y10–Y34 within a follow-up period	Random forest	Fasting plasma glucose, total cholesterol, hemoglobin, aspartate transaminase, alanine transaminase, gamma-glutamyl transpeptidase	Depressive disorder diagnosis, psychopharmacological prescriptions	Income level, type of medical insurance
Ge et al. (64)	Identifying Suicidal Ideation Among Chinese Patients with Major Depressive Disorder: Evidence from a Real-World Hospital-Based Study in China	Neuropsychiatric Disease and Treatment	2020	Retrospective cross-sectional study design	MDD patients from the West China Hospital of Sichuan University	Suicidal ideation measured by item 3 of the Hamilton Depressive Rating Scale	Neural network	Measures of hypothalamic–pituitary-thyroid axis activity	Depressive and anxiety symptoms measured by their respective Hamilton Rating Scales	Marital and vocational status
Haines-Delmont et al. (70)	Testing Suicide Risk Prediction Algorithms Using Phone Measurements With Patients in Acute Mental Health Settings: Feasibility Study	JMIR mHealth and uHealth	2020	Recruitment-based cross-sectional study design	Service users admitted to adult mental health wards in the North West of England, United Kingdom	Suicide risk measured by the Columbia-suicide severity rating scale (C-SSRS)	K-nearest neighbors, random forest, support vector machine	Sleep monitoring	Journaling, safety plan, and mood meter	Step count and frequency and social interaction data via Facebook

Of the 20 selected studies, eight (51–58) investigated suicide-related outcomes in the general population [including two studies in youth (51, 52) and two in elderly (57, 58)]. Six studies (59–64) focused on individuals with a diagnosis of major depressive disorder (MDD) or mood-related disorders [two recruited from psychiatric hospitals (61, 63), one of antidepressant users recruited from either the Australian Genetics of Depression Study or through the nationwide Pharmaceutical Benefits Scheme database (62)], two from the general population (i.e., UK Biobank participants that voluntarily enrolled in the study, and participants of the Brazilian Longitudinal Study of Adult Health, consisting of public institutions’ employees) that met criteria for common mental disorders (59, 60), one from retrospective analysis of health record data (64), and one (65) focused on individuals diagnosed with multiple sclerosis. Five studies (66–70) investigated individuals who were in contact with mental health services [two for emergency department visits (66, 68), and the remaining for substance-related services (67), general help-seeking (69), and admission to mental health wards (70)]. Several of the 20 selected studies made use of more than one ML methodology, with the most popular approach being random forest classification (present in nine studies). In this review, studies are first summarized according to study population – emphasizing types of measures used, top results, and the selected integrative methodology – and then observations are collected and synthesized within the Discussion.

### 3.1 General population studies

#### 3.1.1 Early life (*n* = 2)

Joo et al. (51) assessed *n* = 6,592 children aged 9 to 10 from the Adolescent Brain and Cognitive Development (ABCD) study (71). Multivariate logistic regression, random forest, and elastic net regression were used to predict active, passive, and overall SI as well as SA, measured by the Kiddie Schedule for Affective Disorders and Schizophrenia (K-SADS). The authors assessed feature importance in the elastic net model and highlighted the importance of factors derived from the Child Behavior Checklist (CBCL), which probes problematic behavior as well as depressive or internalizing symptoms. The authors also report that interactions between early life stress and polygenic risk for autism spectrum disorder (ASD) were associated with active and overall suicidal ideation, as well as with overall suicidal thoughts and behaviors, suggesting that genetic predisposition to ASD in consort with early life stress may increase suicidal thoughts and behaviors. However, this interaction effect was not observed in follow-up sex-stratified analyses.

Van Velzen et al. (52) assessed *n* = 5,885 children also from the ABCD study (71). Binomial penalized logistic regressions were used to predict either parent- or child-reported suicidal thoughts and behaviors, recorded in the K-SADS. The authors performed a feature selection procedure applying a ridge penalty to determine the top contributing features. Features that predicted child-reported suicidal thoughts and behaviors were: family conflict, prodromal psychotic symptoms, impulsivity, and the CBCL depression subscale score. Features that predicted parent-reported suicidal thoughts and behaviors were: CBCL depression subscales, CBCL conduct disorder subscale score, CBCL internalizing and externalizing scores, and a history of mental health service use or treatment. Intriguingly, none of the biological features derived from functional neuroimaging or genetics were retained following feature selection. The authors also demonstrated modality-specific classifications, wherein models built with clinical psychiatric, physical health, cognitive functioning, and social–environmental factors each performed well, in contrast to models developed on neuroimaging, sociodemographic, and genetic factors, supporting the results from the feature selection procedure that considered all the factors collectively.

#### 3.1.2 Midlife (*n* = 4)

Allesøe et al. (53) assessed *n* = 65,535 individuals from the Lundbeck Foundation Initiative for Integrative Psychiatric Research (iPSYCH) 2012 database. A feed-forward neural network was developed to predict both SA and completed suicide. When assessing feature importance, the authors found that a set of 516 individual genotypes, selected from published genome-wide association studies of various mental disorders and general medical conditions, were highly important features for both SA and completed suicide. Psychiatric disorders were also equally important for predicting SA, indicating shared genetic architecture between the two, even in the context of family diagnosis history, age, polygenic risk, and medical birth registry information.

Wang et al. (54) assessed *n* = 4,683 individuals from the UK Biobank (72) for short-term (i.e., <1 year) suicide risk prediction, and *n* = 16,660 individuals for long-term (i.e., 1 to 6 years) risk prediction. A light gradient-boosting machine with balanced bagging was developed for risk prediction. The authors developed a polygenic risk score for suicidality but chose to exclude the score in their model development procedure, citing inaccessibility of genetic data outside of the research context. Instead, the authors validated their psychosocial models in a suicide risk-stratified manner, defining low-, moderate-, and high-risk with polygenic score tertiles. The authors noted that mental health-related factors (i.e., history of psychiatric disorders, history of suicide attempt) were consistently important for both long- and short-term suicide risk, but health-related factors were more important for the former and lifestyle and social factors (i.e., responses to the questions: “How many years of using a mobile phone at least once per week to make or receive calls?” and “Age you first had sexual intercourse”), for the latter, indicating temporally relevant risk factors that may differentiate risk trajectories.

Tate et al. (55) assessed a combined *n* = 8,676 individuals from both the Child and Adolescent Twin Study in Sweden (73) and the Netherlands Twin Register (74). Gradient boosted machines, random forest, elastic net, and neural network models were developed to predict suicidal behaviors ascertained from the Life History of Aggression Checklist (for the Swedish cohort) and the Young Adult Self Report and Adult Self Report of the Achenbach System of Empirically Based Assessment (for the Dutch cohort) at age 18. Self-reported aggression, biological sex, and externalizing and internalizing symptoms during adolescence were consistently identified as important features for both the gradient boosted machines and the random forest classifiers. Biological features, such as polygenic risk scores (specifically for general psychopathology, anxiety, and anorexia nervosa), were also important, but relatively less so than included psychosocial factors, such as aggression.

Cho et al. (56) assessed *n* = 372,813 individuals with data available from the health screening cohort database provided by the National Health Insurance Sharing Service of Korea (75). Random forest classifiers were developed to predict individuals who have died by suicide within a specific tracking period, as indicated by an International Classification of Disease (ICD)-10 code of X60–X84 or Y10–Y34. The authors identified physical and lifestyle factors as important features, namely amount of exercise, alcohol intake, and gamma-glutamyl transferase levels, which is a biomarker for liver function and related to alcohol consumption.

#### 3.1.3 Late life (*n* = 2)

Lozupone et al. (57) assessed *n* = 1,252 elderly (aged 65 and above) community-dwelling individuals from the Salus in Apulia Study. A random forest classifier was developed to predict suicidal ideation measured by the Columbia-Suicide Severity Rating Scale (C-SSRS). When considering the entire feature space, education followed by age and diagnosis of mild cognitive impairment were the most important features, indicating that psychosocial factors may play a greater role than biological factors in suicide prediction. The authors also investigated models developed using only the clinical, serum, and genetic biomarkers, and identified the following features as most important: interleukin 6, tumor necrosis factor-alpha, and Apolipoprotein E ε4-carrier status.

Cho et al. (58) assessed *n* = 48,047 elderly (aged 65 and above) individuals with data available from the health screening cohort database provided by the National Health Insurance Sharing Service of Korea (75). Random forest classifiers were developed to predict individuals who have died by suicide, as indicated by ICD-10 codes of X60–X84 or Y10–Y34. Pharmacological factors, namely use of benzodiazepines and sleeping pills, were most important in predicting completed suicides, with gamma glutamyl transpeptidase levels being the most important blood biochemical measure.

### 3.2 Clinical population studies

#### 3.2.1 Depressive disorders (*n* = 6)

Lyall et al. (59) assessed *n* = 19,389 individuals from the UK Biobank (72) that met criteria for broad major depression, defined via the following: analysis of the baseline and follow-up mental health questionnaires, ICD-10 codes for mood disorders (F32-F34, F38, F39), and nurse-led interviews. This population was then further subdivided based on indication of suicidal thoughts and behaviors present on the included questionnaires. Logistic regression, as well as least absolute shrinkage and selection operator (LASSO), ridge, and elastic net penalized regression models were developed to predict group status. Sleep-derived biological features such as mean duration of sustained inactivity bouts during the daytime and self-reported insomnia symptoms were found to be the most important features in the ridge penalized predictive model.

Roza et al. (60) assessed *n* = 4,039 individuals from the Brazilian Longitudinal Study of Adult Health (76). Individuals were assessed at baseline with the Brazilian Portuguese version of the Clinical Interview Schedule-Revised (CIS-R). Individuals identified as having common mental disorders (CIS-R score > 12), namely anxiety and depression, were included in this study. Elastic net regularization, random forest, Naïve Bayes, and ensemble (combining the first three) classifiers were developed to predict suicide risk measured by three specific questions from the CIS-R probing hopelessness, passive SI, or active SI. Features relating to depression and anhedonia, particularly in the last seven days, were identified as important predictors regardless of model architecture.

Campos et al. (62) assessed *n* = 10,413 individuals from the Australian Genetics of Depression Study (77) with no reported suicidality before antidepressant treatment. Naïve Bayes, decision tree, adaptive boosting, random forests, and logistic regression models were developed to predict treatment-attributed suicidal ideation (TASI), self-reported on the Antidepressants Efficacy and Side Effects Questionnaire. The authors found no genome-wide significant risk loci for TASI and thus did not include any biological predictors in their final models. Instead, the authors highlight strong associations with comorbid psychiatric diagnoses (such as personality disorder, bipolar disorder, and post-traumatic stress disorder), and with thoughts of death and changes in appetite during past depressive episodes.

Ge et al. (64) assessed *n* = 1,916 patients with major depressive disorder (MDD) from the West China Hospital of Sichuan University. A neural network was developed to predict suicidal ideation as measured by item #3 on the HAM-D. Network weights identified free thyroxine, total HAM-D scores, and vocational status as the most relevant predictors.

Li et al. (63) assessed *n* = 1,718 patients with first-episode, untreated MDD recruited from the Department of Psychiatry, First Clinical Medical College of Shanxi Medical University in Taiyuan, China. Patients were then interviewed and grouped into one of three categories based on lifetime history of suicide attempt: non-attempters, recent attempters (within two weeks of assessment), and long-dated attempters (having attempted more than a month prior to assessment). Separate gradient boosted decision tree models were developed with extreme gradient boosting to predict both recent and long-dated attempters. Across both models, hostility as measured by the Positive and Negative Syndrome Scale for Schizophrenia (PANSS) and total score on the HAM-A were identified as top features. Excitement as measured by the PANSS and total score on the HAM-D were also important for classifying recent attempters. Marriage status, specifically being single, was important for long-dated attempters. Regarding biological predictors, free thyroxine was the most important for the former, and low-density lipoprotein cholesterol for the latter.

In the same Chinese cohort, Yang et al. (61) categorized the patients into suicide non-attempters and attempters with no temporal distinction. LASSO regression models were developed to screen 20 input features based on feature importance, with seven features surviving: psychotic and anxiety symptoms, anti-thyroglobulin, thyroid peroxidase antibodies, serum total cholesterol, high-density lipoprotein cholesterol, and subclinical hypothyroidism.

#### 3.2.2 Multiple sclerosis (*n* = 1)

Barak-Corren et al. (65) assessed *n* = 15,117 patients diagnosed with multiple sclerosis (ICD-9 of 340) registered with the Partners Healthcare Research Patient Data Registry (RPDR). A Naïve Bayesian classifier was developed to predict suicidal behavior, as defined by a series of ICD-9 codes recorded in either the RPDR, or in death certificates from the Commonwealth of Massachusetts. The authors do not explicitly investigate relative feature importance of their derived risk scores, but did highlight condition-specific predictors, such as chronic pain, as being important for the prediction of suicidal behavior in patients with multiple sclerosis.

#### 3.2.3 Emergency department visits (*n* = 2)

Edgcomb et al. (66) assessed *n* = 1,713 youth aged 10–17 years who presented in an emergency department (ED) for mental health–related reasons. After sampling to ensure an equal distribution of 50% cases for suicide-related ED visits and 50% non-suicide cases, the authors developed LASSO regression and random forest classifiers to predict the suicide-related cases. The only feature important to the predictive ability of both the LASSO and random forest models (aside from the features used in the group definitions for the suicide-related cases, which the authors included in their feature space) was and ICD-10 diagnosis of depressive disorders. ICD-10 codes for trauma- and stressor-related disorders were important for the former, and National Area Deprivation Index for the latter, indicating different risk profiles dependent on the ML architecture chosen. Blood laboratory test measures included in the models were not among the selected features based on feature importance.

Grendas et al. (68) assessed *n* = 308 patients admitted to an emergency department in Argentina for active suicidal ideation or a recent suicide attempt. The authors performed survival analyses on subsequent suicides or reattempts as the outcome, ascertained by follow-ups with the patient at 6, 12, 18, and 24 months after admission. Cox regression models and random survival forests were both developed. Consistently across all feature importance investigations (backward elimination for Cox regression, and positive importance and minimum depth methods for the random survival forests), the 5-HTTLPR polymorphism—found in SLC6A4, the gene that codes for the serotonin transporter—was shown to be important.

#### 3.2.4 Care-seeking (*n* = 3)

Balbuena et al. (67) assessed a subset of *n* = 173,275 individuals from the Cohort of Norway (78) and *n* = 12,614 individuals that visited a Saskatoon hospital for a mental health or substance-related reason between 2011 and 2016. Similar to Grendas et al. (68), the authors performed survival analyses on subsequent completed suicide ascertained through ICD-10 codes X60-X84 and Y87.0 recorded in either the Norwegian Causes of Death Registry, or by the Saskatchewan provincial coroner. The authors also developed sex-specific Cox regression models and random survival forests and determined for the Cohort of Norway that resident income status, smoking-related exposures, and mood symptoms to be important predictors shared across both sex-specific models. None of the biological measurements (triglycerides, HDL-cholesterol, glucose, total cholesterol) were identified as important predictors. In the Saskatoon models, only age and sex were found to be important.

Oppenheimer et al. (69) assessed *n* = 84 help-seeking young adults aged 18–25 years recruited from the general public. The authors defined help-seeking broadly as soliciting of any mental health-related counseling or psychiatric service. Suicidal thoughts and behaviors were measured by the 6-item suicidality subscale of the Mood Spectrum Self-Report questionnaire. Similar to Yang et al. (61), LASSO regression was used to reduce the input feature space to only important and contributing predictors of suicidality. Five features survived the feature reduction process, including age, level of education, depressive symptoms, and psychological distress. One feature derived from functional MRI was also retained: activation of left amygdala while observing sad faces.

Haines-Delmont et al. (70) assessed *n* = 66 individuals admitted to adult mental health wards across the North West of England, United Kingdom. Suicidal thoughts and behaviors of participants were assessed with the Columbia-suicide severity rating scale and predicted using K-nearest neighbor, random forest, support vector machine, and logistic regression. Unfortunately, social interaction data captured via social media usage was sparse due to lack of permission granted and access issues and was not included in the final analyses. From analyses of entropy in the random forest models, the authors identify journal feeling, or sentiment derived from freeform journal entries, as the most important feature, followed by time spent in bed.

### 3.3 Risk of bias assessment

The risk of bias has been assessed in all 20 studies, with twelve being identified by both reviewers (EST and MCM) as having low risk of bias. Three studies were assessed as having some concerns of bias, and five as having high risk of bias (Supplementary Tables S2, S3). Studies categorized as having high risk of bias demonstrated biases in the selection of their target populations, often involving either single-center/region or non-random sampling methods. These studies also used questionnaires or survey instruments that lacked reliability and had not been previously validated in other cohorts. Cohen’s kappa was 0.66, indicating moderate agreement between both reviewers. We have presented both raters’ assessments in separate columns of Supplementary Tables S2, S3.

## 4 Discussion

We systematically reviewed 20 studies of suicide-related outcomes, each taking a biopsychosocial, Whole Person approach and using ML methods. Of these 20, eight studies were conducted in general populations and 12 were conducted in clinical populations.

### 4.1 Heterogeneity in study outcomes

A key challenge observed among studies reviewed was that measured suicide-related outcomes were heterogeneous – modeled using different tools and definitions – despite a growing literature on operational distinctions between types and degrees of suicidal thoughts and behaviors (e.g., ideation vs. attempt vs. completion) (79, 80). This is partly attributable to the enduring prevalence of scales that do not offer a degree of specificity matching modern theory (81–83). Accurate terminology for suicide-related outcomes is required for future studies to investigate individual components of suicide.

In this review, the 20 selected studies ascertained suicide-related outcomes through (1) ICD codes, (2) external assessments such as by clinician chart reviews, (3) patient self-reports, or (4) via interviews using one of eight distinct questionnaires. Such a variety of outcome measures makes it difficult to draw conclusions on the overall landscape of risk and predictive factors since each questionnaire could be operationalizing a different aspect of suicidal thoughts and behaviors. For example, the C-SSRS (84) assess risk behavior and lethality of the suicide risk and was developed as a suicide screening tool. Another tool used for suicide screening is the HAM-D (43), specifically item three on the HAM-D. Available responses range from “Absent,” to “Feels life is not worth living,” to “Wishes he/she were dead,” to “Ideas or gestures of suicide,” and most severely, “Attempts at suicide.” Unlike the BSI, item three on the HAM-D does not account for behaviors preceding the moment of screening, nor is it comprehensive in terms of the number of questions asked. Thus, in this review we compare predictors within a study, constrained to the outcome defined by the authors and instead discuss trends of consistent biopsychosocial factors across studies of different outcomes. This issue may be addressed more systematically in research by harmonization initiatives, such as the National Institute of Mental Health (NIMH) Common Data Elements (CDE), which is a minimal list of data collection instruments – developed with the Wellcome Trust and other international funding agencies – to be used in all NIMH-funded clinical research moving forward (85). However, the CDE list does not include instruments designed for the measure of suicidal thoughts and behaviors.

### 4.2 Evidence for the biopsychosocial model of suicide

We assessed evidence for the biopsychosocial model of suicidal thoughts and behaviors by the evaluating the relative performance of ML models included in our reviewed studies and how they were affected by the inclusion or exclusion of variables across different domains. The majority of studies (51, 54–56, 58, 60, 63, 64, 67, 70) address this “value add” question as a matter of relative variable importance, in a post-hoc manner, where explicit variable priority measures are calculated [such as SHapley Additive exPlanations (SHAP) (86)] or derived feature weights are directly compared. Another approach used by some studies in this review (52, 59, 61, 68, 69) is to reduce the total feature space through selection methods such as penalized regression. This approach identifies features with non-zero penalized weights as important features and provides insight into the relative importance of a variable within the entire feature space. Demographic and psychological variables are often identified as the top predictors in these approaches, followed by biological measures like polygenic risk scores. The relative impact of each individual feature, however, cannot be assessed and the definition of an “important feature” across studies is not fixed or entirely consistent. In the absence of consistent, comparative benchmarks, we chose to apply reasonable thresholds (i.e., on beta values in the case of penalized regressions, or on the rankings of feature importance statistics when no thresholds were provided) when defining the top predictors of each study. In the case where a feature selection procedure was used by the source study, we report all retained features as important features (i.e., features with non-zero beta coefficients resulting from a LASSO regression model) in Supplementary Table S4. However, when only feature importance statistics were provided for the full set of features, we chose a rank-based approach to define “important features.” Crucially, to account for variability in the number of features considered by different studies, we chose to apply a percentile-based thresholding approach, selecting the top decile of features as “important.” This ensured a proportional number of variables listed as “important” across studies. However, it should be noted that this approach was only valid when the total number of features used in a given model was provided by the source study. In the cases where it was not, we chose to list the top three variables as the most “important.”

A few studies were more rigorous in their approach. For example, Allesøe et al. (53) investigated the biopsychosocial model directly by calculating feature importance separately for each data type: family diagnosis history, age, individual genotype data, polygenic risk score, mental disorder diagnosis, and medical birth registry. The authors assessed overall feature importance by then removing all variables in each data set iteratively and retraining the models at each step, deriving relative drops in model accuracy as a measure of importance. This approach demonstrates the incremental value of a biopsychosocial model, and the authors found the impact of individual genetics to be comparable to that of psychiatric diagnoses, providing evidence for the biopsychosocial model. Edgcomb et al. (66) developed and compared three separate models built on varying levels of health record data: (1) mental health–related ICD-10-CM codes and mental health–related chief complaints (34 features); (2) suicide-related ICD-10-CM codes and all child sociodemographics and clinical characteristics (including laboratory tests), excluding mental health–related ICD-10-CM codes (53 features); and (3) all structured data elements (84 features). This approach allows for the comparison of ML metrics, such as sensitivity, specificity, and accuracy, as a function of the feature space. The authors found consistent results across all three sets of data, with the third set performing better or at least as well as the first set, and the second set performing the worst. The authors did not find any substantial increases in predictive power with the inclusion of more data, and post-hoc feature importance analyses also show that ICD-10 codes for comorbid psychiatric disorders (included in the first set) as the most important features. Lozupone et al. (57) performed a similar investigation to Edgcomb et al. (66), wherein the authors developed separate models built on different sets of data. In models of only clinical, serum, and genetic biomarkers, the authors identified inflammatory markers interleukin 6 and tumor necrosis factor-alpha as important predictors. However, in the final model including all selected predictors, post-hoc feature importance analyses identified education, age, mild cognitive impairment, and gender as the most important contributors instead, superseding the biological features in relative importance.

Overall, evidence from our reviewed literature provides some support for the integration of multi-domain data, particularly biological signals, though this support is not universal. Predictive models of suicidal thoughts and behaviors are strongly driven by psychological factors such as comorbid psychiatric illness. However, some biological signals can be observed, with genetic markers showing more potential over circulating fluid markers. At this early juncture in the field, definitive conclusions are not possible. More studies including a wider range of biological markers (i.e., those underrepresented in integrative studies: gene expression signatures, neuroimaging-derived features, and electrophysiological measures) and their integration with psychosocial measures are needed to continue filling in our empirical picture of the biopsychosocial model of suicide.

### 4.3 Consistent biopsychosocial indicators and predictors

**Figure 2:**
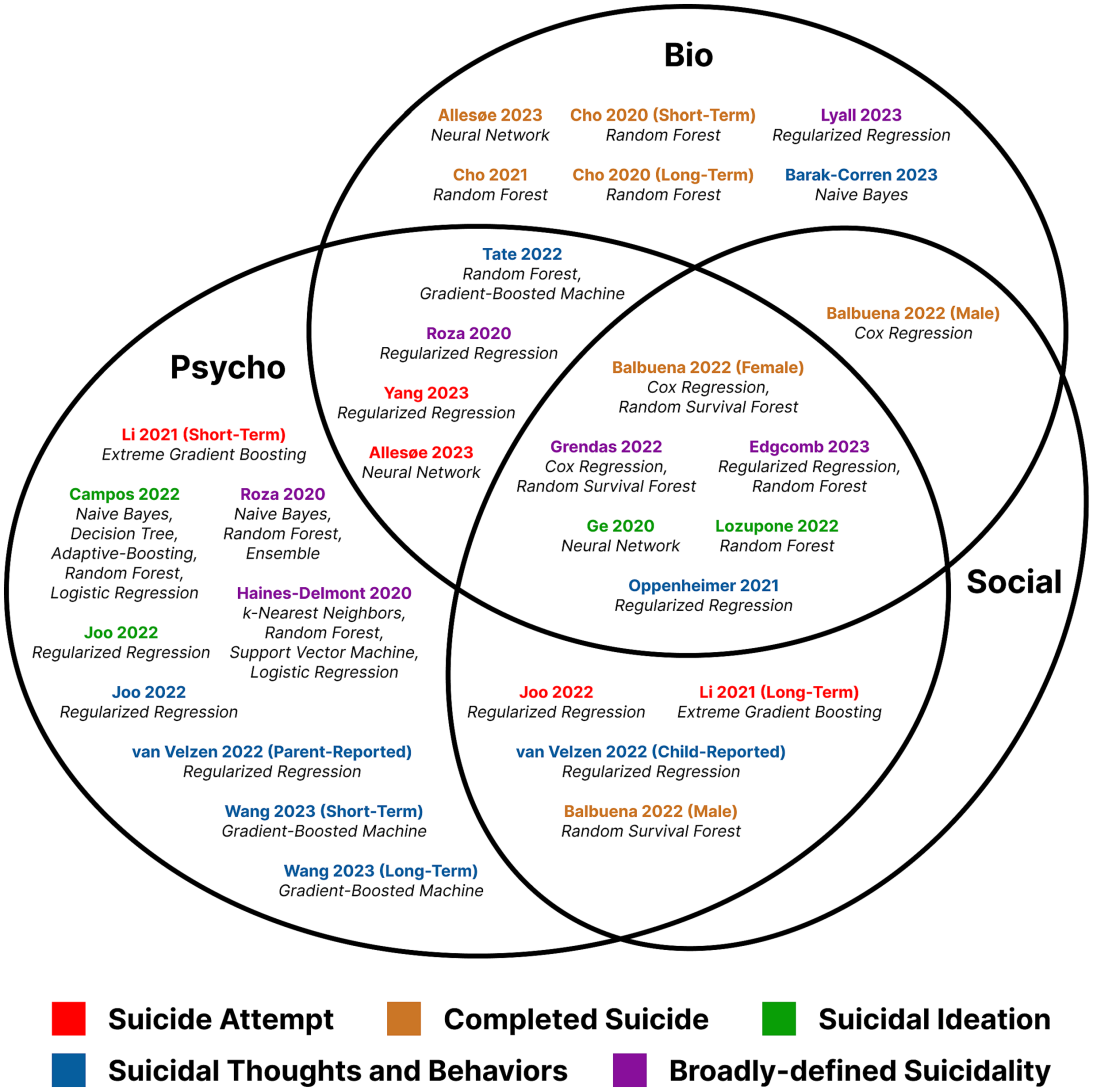
Venn diagram of included studies categorized by most important features, selected by rank-based thresholding of the feature importance metric or by feature selection algorithms.

We assessed the top predictive features of each ML model and categorized them into one of seven possible combinations of the three domain types (“bio,” “psycho,” and “social”). Despite heterogeneity in study design, suicide outcome, and model architecture, we were able to identify several features and types of features that integrative ML models identified more or less consistently as important for suicide phenotypes (Figure 2). However, it is important to note that only one study, Campos et al. (62), externally validated their identified predictors in an independent cohort. This lack of external validation in the other studies can limit the generalizability of the model-derived insights. From the biological domain, measures of hypothalamic–pituitary-thyroid (HPT) axis activity were identified by three separate studies (61, 63, 64) as predictive of suicidal thoughts and behaviors in patients with MDD. Recent work has shown involvement of the HPT axis with dopaminergic systems in the pathophysiology of suicidal behavior (87). However, all three studies were from psychiatric hospitals in Chinese populations, with two studies overlapping in their investigations of the same cohort, limiting conclusions on generalizability of these findings. Other studies including fluid biomarkers measured with standard laboratory tests did not find them to be more informative than proximal psychological measures (60), comorbid psychiatric diagnoses (66), or sociological factors like income levels (67).

Findings regarding genetic predictors of suicide in our reviewed studies were inconsistent across clinical and general population studies. A GWAS conducted by Campos et al. (62) found no genetic signal for treatment-attributed suicidal ideation. In models of completed suicide, Grendas et al. (68) identified the serotonin-transporter-linked promotor region (5-HTTLPR) polymorphism as a significant predictor. The discrepancy in findings could be due to postulated distinct genetic architectures underlying completed suicides and suicidal ideation (88), further complicated by the distinction of treatment-related ideation. Furthermore, Grendas et al.’s targeted genotyping approach is not subjected to the same statistically stringent constraints applied to a GWAS. Similarly, in the general population studies, particularly of mid-life, individual genotypes were identified as important predictors for both suicide attempt and completed suicide (53). Whereas polygenic risk scores for suicidality were not predictors of short- or long-term suicide risk (54). Polygenic risk scores for psychiatric disorders like anxiety and anorexia nervosa were shown to be important for predicting suicidal behaviors (55). No clear genetic marker for suicide has been established, and further work exploring candidate gene approaches through a biopsychosocial lens would be beneficial.

In studies of early life suicidal behaviors, psychosocial factors dominated feature importance rankings. Namely, variables relating to behavior and internalizing and externalizing symptoms were much more important that genetic or neuroimaging derived features (51, 52). However, these are results from only one cohort, the ABCD study, so more studies of suicide-related behaviors in young children are needed to better inform the variability of risk profiles across the lifespan. On the other hand, in studies of late life suicidal behaviors, the most salient predictors are generally age-related, such as dementia and pharmacological dependence (57, 58). Some other interesting biological features identified in this review include sleep, both objectively measured and subjectively experienced. Sleep-related features were consistently ranked highly in terms of feature importance when included in models of suicide within a clinical population (59, 70). As more large-scale, multi-modal human population studies become available and gain popularity, consensus may emerge regarding the relative importance of these features when considered among their biopsychosocial counterparts in Whole Person models.

Finally, the only neuroimaging-derived features tested in any study qualifying for review were derived from fMRI (69), and the most important feature was determined to be activity in the left amygdala while observing sad faces. Despite ample evidence supporting detectable inter-individual variability in neuroimaging studies of suicide-related phenotypes (89, 90), imaging-derived variables are not often integrated with other variables in a Whole Person approach to modeling. The majority of “bio”-only studies excluded from this review (see Figure 1) are neuroimaging studies that focus solely on imaging-derived features, such as higher dimensional data (i.e., whole image and voxel analyses), or functional connectivity values. Similarly, another emerging field in the prediction of suicide-related outcomes using ML is natural language processing (NLP). Studies of this type use large corpuses of unstructured data, ranging from clinician notes (the psychological domain) or social media posts (the sociological domain) to even audio recordings of psychiatric interventions. NLP is particularly well-suited for early detection and prevention of mental health disorders, due to the immediacy and convenience of social media (91). However, its utility in predictive modeling is limited similarly to neuroimaging studies in that the context surrounding the text (or image for the latter) is not captured unless integrative modeling is applied to augment domain-specific features. For example, specific patterns of speech or text may have different meanings for individuals with different innate stress responses, which are to some degree influenced by genetics (92).

## 5 Conclusion

We identified 20 studies of human general and clinical populations that developed ML models of suicide-related outcomes including features across a transdisciplinary combination of biological, psychological, and sociological data types – Whole Person Models. The majority of studies identified psychological features, such as depressive symptoms and comorbid psychiatric diagnoses, as most important for predicting suicide-related outcomes. Genetic features, such as individual genotypes and polygenic risk scores, had variable impact on model performance depending on the outcome. Other biological features, such as objective sleep measures, functional neuroimaging, and hypothalamic–pituitary-thyroid axis markers showed predictive ability in the presence of other psychosocial factors. More accurate and specific definitions of suicidal behaviors will permit better study designs for risk factor identification and suicide prediction. The sum of evidence suggests that future studies could benefit from the inclusion of biopsychosocial data in their predictive models, though more in-depth analyses of feature importance and greater breadth of biological characterizations are needed.

## Data availability statement

The original contributions presented in the study are included in the article/Supplementary material, further inquiries can be directed to the corresponding author.

## Author contributions

ET: Conceptualization, Formal analysis, Methodology, Writing – original draft. MM: Formal analysis, Methodology, Writing – original draft. DF: Conceptualization, Funding acquisition, Supervision, Writing – review & editing.
