# Supplementary material for: Evidence for the biopsychosocial model of suicide: a review of whole person modeling studies using machine learning
Source: Front Psychiatry. 2024 Jan 11;14:1294666. doi: 10.3389/fpsyt.2023.1294666 (PMC10808719; doi:10.3389/fpsyt.2023.1294666)
Supplement: Supplementary file 1 [file Table_1.DOCX]

Supplementary Data

**Evidence for the Biopsychosocial Model of Suicide: A Review of Whole Person Modeling Studies Using Machine Learning**

Earvin S. Tio BASc^1,2^, Melissa C. Misztal MHSc^1^, Daniel Felsky PhD^1,2,3,4,*^

1. Krembil Centre for Neuroinformatics, Centre for Addiction and Mental Health, Toronto, ON

2. Institute of Medical Science, University of Toronto, Toronto, ON

3. Biostatistics Division, Dalla Lana School of Public Health, University of Toronto, Toronto, ON

4. Department of Psychiatry, University of Toronto, Toronto, ON

*Corresponding Author: Daniel Felsky PhD

Krembil Centre for Neuroinformatics,

Centre for Addiction and Mental Health, 12^th^ Floor, 250 College Street

Toronto, ON, M5T 1R8

Email: [Daniel.felsky@camh.ca](mailto:Daniel.felsky@camh.ca)

Alternate email: [dfelsky@gmail.com](mailto:dfelsky@gmail.com)

Phone: 416-939-0423

Keywords: suicide, suicidal ideation, machine learning, whole person, review

**Table 1:** Search terms used to identify records across the five electronic databases considered.

| **Electronic Database** | **Search Terms** |
| --- | --- |
| EMBASE | (suicid*) and (“machine learning” or “artificial intelligence” or “deep learning”) and (predict* or classif*)).mp. [mp=title, abstract, heading word, drug trade name, original title, device manufacturer, device trade name, keyword heading word, floating subheading word, candidate term word] |
| MEDLINE | (suicid*) and (“machine learning” or “artificial intelligence” or “deep learning”) and (predict* or classif*)).mp. [mp=title, book title, abstract, original title, name of substance word, subject heading word, floating sub-heading word, keyword heading word, organism supplementary concept word, protocol supplementary concept word, rare disease supplementary concept word, unique identifier, synonyms, population supplementary concept word, anatomy supplementary concept word] |
| PsychINFO | (suicid*) and (“machine learning” or “artificial intelligence” or “deep learning”) and (predict* or classif*)).mp. [mp=title, abstract, heading word, table of contents, key concepts, original title, tests & measures, mesh word] |
| PubMed | "suicid*"[All Fields] AND ("machine learning"[MeSH Terms] OR ("machine"[All Fields] AND "learning"[All Fields]) OR "machine learning"[All Fields] OR ("artificial intelligence"[MeSH Terms] OR ("artificial"[All Fields] AND "intelligence"[All Fields]) OR "artificial intelligence"[All Fields]) OR ("deep learning"[MeSH Terms] OR ("deep"[All Fields] AND "learning"[All Fields]) OR "deep learning"[All Fields])) AND ("predict*"[All Fields] OR "classif*"[All Fields]) |
| Web of Science | ((ALL=(suicid*)) AND ALL=((“machine learning”) OR (“artificial intelligence”) OR (“deep learning”))) AND ALL=((predict*) OR (classif*)) |

**Table 2:** Risk of bias assessment using a modified version of the Risk of Bias Instrument for Cross-Sectional Surveys of Attitudes and Practices.

| **Authors** | **Title** | **EST bias rating** | **Risk of bias** | **MCM bias rating** | **Risk of bias** | **Overall risk of bias** |
| --- | --- | --- | --- | --- | --- | --- |
| Allesøe et al. | Deep Learning for Cross-Diagnostic Prediction of Mental Disorder Diagnosis and Prognosis Using Danish Nationwide Register and Genetic Data | 4 | low | 5 | low | low |
| Barak-Corren et al. | Improving risk prediction for target subpopulations: Predicting suicidal behaviors among multiple sclerosis patients | 8 | high | 10 | high | high |
| Cho et al. | Development of a Suicide Prediction Model for the Elderly Using Health Screening Data | 7 | low | 7 | low | low |
| Cho et al. | Prediction of suicide among 372,813 individuals under medical check-up | 6 | low | 13 | high | moderate |

High-risk is defined as a score >7. Overall risk is determined by concordance of the individual assessments.

**Table 3:** Risk of bias assessment using the Risk of Bias Instrument for Cross-Sectional Surveys of Attitudes and Practices.

| **Authors** | **Title** | **EST bias rating** | **Risk of bias** | **MCM bias rating** | **Risk of bias** | **Overall risk of bias** |
| --- | --- | --- | --- | --- | --- | --- |
| Edgcomb et al. | Assessing Detection of Children With Suicide-Related Emergencies: Evaluation and Development of Computable Phenotyping Approaches | 14 | high | 14 | high | high |
| Lyall et al. | Subjective and objective sleep and circadian parameters as predictors of depression-related outcomes: A machine learning approach in UK Biobank | 13 | high | 12 | low | moderate |
| Roza et al. | Suicide risk classification with machine learning techniques in a large Brazilian community sample | 8 | low | 9 | low | low |
| Wang et al. | Prediction of Suicidal Behaviors in the Middle-aged Population: Machine Learning Analyses of UK Biobank | 13 | high | 15 | high | high |
| Yang et al. | Establishment of a risk prediction model for suicide attempts in first-episode and drug nai¨ve patients with major depressive disorder | 8 | low | 15 | high | moderate |
| Balbuena et al. | Identifying long-term and imminent suicide predictors in a general population and a clinical sample with machine learning | 9 | low | 11 | low | low |
| Campos et al. | Clinical, demographic, and genetic risk factors of treatment-attributed suicidality in >10,000 Australian adults taking antidepressants | 10 | low | 12 | low | low |
| Grendas et al. | Comparison of traditional model-based statistical methods with machine learning for the prediction of suicide behaviour | 7 | low | 7 | low | low |
| Joo et al. | Association of Genome-Wide Polygenic Scores for Multiple Psychiatric and Common Traits in Preadolescent Youths at Risk of Suicide | 7 | low | 8 | low | low |
| Lozupone et al. | Apolipoprotein E genotype, inflammatory biomarkers, and non-psychiatric multimorbidity contribute to the suicidal ideation phenotype in older age. The Salus in Apulia Study | 9 | low | 9 | low | low |
| Tate et al. | A genetically informed prediction model for suicidal and aggressive behaviour in teens | 13 | high | 14 | high | high |
| van Velzen et al. | Classification of suicidal thoughts and behaviour in children: results from penalised logistic regression analyses in the Adolescent Brain Cognitive Development study | 7 | low | 9 | low | low |
| Li et al. | Identifying clinical risk factors correlate with suicide attempts in patients with first episode major depressive disorder | 8 | low | 8 | low | low |
| Oppenheimer et al. | Informing the study of suicidal thoughts and behaviors in distressed young adults: The use of a machine learning approach to identify neuroimaging, psychiatric, behavioral, and demographic correlates | 10 | low | 10 | low | low |
| Ge et al. | Identifying Suicidal Ideation Among Chinese Patients with Major Depressive Disorder: Evidence from a Real-World Hospital-Based Study in China | 10 | low | 12 | low | low |
| Haines- Delmont et al. | Testing Suicide Risk Prediction Algorithms Using Phone Measurements With Patients in Acute Mental Health Settings: Feasibility Study | 17 | high | 14 | high | high |

High-risk is defined as a score >12. Overall risk is determined by concordance of the individual assessments.

**Table 4:** Model performance and feature importance metrics extracted from the final set of reviewed studies.

| **Study** | **Outcome** | **Machine Learning** | **Validation** | **Model Performance** | **Important Predictors*** | **Total # of predictors** |
| --- | --- | --- | --- | --- | --- | --- |
| Allesøe et al., 2023 | Suicide | Feed-forward neural network | 10% of the sample was used for hyperparameter selection with 3-fold cross-validation | AUC = 0.48, accuracy = 99.84% | Individual genotypes: reduction in prediction accuracy = 1.98 (1.51, 2.71) % | 9 categories of predictors |
|  | Suicide attempt | Feed-forward neural network |  | AUC = 0.90, accuracy = 81% | Individual genotypes: reduction in prediction accuracy = 10.27 (9.25, 11.50) % Psychiatric disorders: reduction in prediction accuracy = 10.67 (10.54, 10.81) % | 9 categories of predictors |
| Barak-Corren et al., 2023 | Suicidal behavior | Naïve Bayesian classifier | Internal validation was conducted with a testing subset that is half the size of the cohort | ROC = 0.77, specificity = 90%, sensitivity = 0.37, PPV = 0.02, NPV = 1 | Other and unspecified noninfectious gastroenteritis and colitis: OR = 4.5 (distance from the mean in SDs = 1.35) Other chronic pain: OR = 4.3 (distance from the mean in SDs = 1.35) Brachial neuritis or radiculitis NOS: OR = 4 (distance from the mean in SDs = 1) | Total number of predictors not explicitly stated |
| Edgcomb et al., 2023 | Suicide-related cases | Lasso regression | Internal validation was conducted with 10-fold cross-validation | Sensitivity = 0.86, specificity = 0.91, accuracy = 0.88 | ICD-10: depressive disorders: median coefficient from cross-validation = 0.87 Hispanic: median coefficient from cross-validation = 0.84 Antihistamines: median coefficient from cross-validation = -0.81 Prior 365 day hospitalization: median coefficient from cross-validation = -0.77 Alcohol: median coefficient from cross-validation = 0.57 Psychiatric admission: median coefficient from cross-validation = 0.56 ICD-10 Group: Mental Health Symptom: median coefficient from cross-validation = 0.47 Injectables: median coefficient from cross-validation = 0.47 | 84 |
|  |  | Random forest |  | Sensitivity = 0.86, specificity = 0.91, accuracy = 0.88 | ICD-10: depressive disorders: median feature importance from cross-validation = 0.05 Psychiatric admission: median feature importance from cross-validation = 0.05 Patient age: median feature importance from cross-validation = 0.03 State Area Deprivation Index: median feature importance from cross-validation = 0.03 National Area Deprivation Index: median feature importance from cross-validation = 0.03 Site: median feature importance from cross-validation = 0.03 Involuntary legal status: median feature importance from cross-validation = 0.02 ICD-10 Group: Other Medical Condition: median feature importance from cross-validation = 0.02 | 84 |
| Lyall et al., 2023 | Suicidality | Ridge penalised regression | Internal validation was conducted with a testing subset that is 25% of the data | Area under ROC curve = 0.63, sensitivity = 50.48%, specificity = 69.35%, accuracy = 68.12% | NA (absolute values of coefficients not reported) |  |
| Roza et al., 2023 | Suicide risk | Elastic net regularization | Internal validation was conducted with nested 10-fold cross-validation | AUC = 0.773, AUC PR = 0.439, sensitivity = 0.747, specificity = 0.659 | Rank by feature importance (top 3): Sad/depressed in last 7 days HRV Triangular index Felt not so good as other people in last 7 days | 30 |
|  |  | Naïve Bayes |  | AUC = 0.798, AUC PR = 0.566, sensitivity = 0.922, specificity = 0.473 | Rank by feature importance (top 3): #days sad/incapable of enjoyment last 7 days Felt not so good as other people in last 7 days Depression symptoms | 18 |
|  |  | Random forest |  | AUC = 0.814, AUC PR = 0.598, sensitivity = 0.630, specificity = 0.792 | Rank by feature importance (top 3): Felt not so good as other people in last 7 days Depression symptoms #days sad/incapable of enjoyment last 7 days | 30 |
|  |  | Ensemble |  | AUC = 0.811, AUC PR = 0.596, sensitivity = 0.899, specificity = 0.510 | Rank by feature importance (top 3): Felt not so good as other people in last 7 days Sad/depressed in last 7 days #days sad/incapable of enjoyment last 7 days | 30 |
| Wang et al., 2023 | Short term suicidal behavior | Light gradient-boosting machine with balanced bagging | Internal validation was conducted with stratified 10-fold cross-validation and further validated in subsets of the cohort with differing genetic susceptibilities | AUC = 0.888, sensitivity = 57.85%, specificity. = 95.11 | Rank by feature importance (top 2) History of psychiatric disorders Have you ever seen a psychologist for nerves, anxiety, tension or depression? | 20 |
|  | Long term suicidal behavior | Light gradient-boosting machine with balanced bagging |  | AUC = 0.852, sensitivity = 54.74%, specificity = 94.05% | Rank by feature importance (top 2) History of psychiatric disorders Have you ever seen a psychologist for nerves, anxiety, tension or depression? | 20 |
| Yang et al., 2023 | Suicide attempt | LASSO regression | Internal validation was conducted with a testing subset that is 30% of the data | AUC = 0.720, threshold probabilities = 20-60% | Anxious symptoms: OR = 5.994 (95% CI = 3.208, 11.197) Psychotic symptoms: OR = 3.572 (95% CI = 2.526, 5.052) Thyroid peroxidase antibodies: OR = 2.061 (95% CI = 1.49, 2.85) Serum total cholesterol: OR = 1.461 (95% CI = 1.081, 1.975) Anti-thyroglobulin: OR = 1.338 (95% CI = 0.936, 1.913) Subclinical hypothyroidism: OR = 1.172 (95% CI = 0.841, 1.633) High density lipoprotein cholesterol: OR = 0.487 (95% CI = 0.309, 0.768) | all retained predictors from penalised regression |
| Balbuena et al., 2022 | Suicide | Cox regression | Internal validation was conducted with a testing subset of unspecified size | AUC calculated at 267 months follow-up: Females = 0.38 | Females: Daily smoking: HR = 1.52 (95% CI = 1.08, 2.14) Mood symptoms: HR = 1.11 (95% CI = 1.02, 1.21) Proportion of county with low income: HR = 1.09 (95% CI = 1, 1.17) | Total number of predictors not explicitly stated |
|  |  |  |  | AUC calculated at 267 months follow-up: Males = 0.57 | Males: Daily smoking: HR = 2.06 (95% CI = 1.21, 3.51) Triglycerides: HR = 1.28 (95% CI = 1, 1.64) Proportion of county with low income: HR = 1.23 (95% CI = 1.08, 1.40) |  |
|  |  | Random survival forest |  | AUC calculated at 267 months follow-up: Females= 0.50 | Females feature rank: 1. Mood symptoms 2. Proportion of county with low income 3. Daily smoking |  |
|  |  |  |  | AUC calculated at 267 months follow-up: Males = 0.43 | Males feature rank: 1. Married 2. Mood symptoms 3. Lives with spouse/partner |  |
| Campos et al., 2022 | Treatment-associated suicidal ideation | Naïve Bayes | Internal validation was conducted with a testing subset that is 33.3% of the data and further external validation was conducted (bi-directionally) with the Australian Genetics of Bipolar Study (AGBS) | AUC = 0.713 | NA (coefficients in the feature selection procedure are reported, but not for post-hoc analysis of the trained models) | NA |
|  |  | Decision tree |  | AUC = 0.573 |  |  |
|  |  | AdaBoost |  | AUC = 0.770 |  |  |
|  |  | Random forest |  | AUC = 0.750 |  |  |
|  |  | Logistic regression |  | AUC = 0.771 |  |  |
| Grendas et al., 2022 | Subsequent suicide or a suicide reattempt | Cox regression | Internal validation was conducted with a testing subset that is 33.3% of the data | AUC = 0.511, sensitivity = 0.000, specificity = 1.000, accuracy = 0.508 | 5-HTTLPR polymorphism (LL vs. SS): HR = 2.2405 (95% CI = 1.7454, 2.7356) Number of previous suicide attempts (>3 vs. 0): HR = 1.8667 (95% CI = 1.3444, 2.3890) Number of previous suicide attempts (1 or 2 vs. 0): HR = 1.7677 (95% CI = 1.2475, 2.2879) 5-HTTLPR polymorphism (SL vs. SS): HR = 1.2232 (95% CI = 0.7638, 1.6826) Age: HR = 0.7326 (95% CI = 0.5323, 0.9329) Partnership/cohabiting: HR = 0.5766 (95% CI = 0.1574, 0.9958) | all retained predictors from feature selection |
|  |  | Random survival forests |  | AUC = 0.799, sensitivity = 0.611, specificity = 0.806, accuracy = 0.704 | Percentage (%) of times retained: 5-HTTLPR polymorphism: 88.2 Age: 84.0 Number of recent stressors: 74.8 Age at first SA: 72.8 Number of previous suicide attempt: 72.6 Child sexual abuse: 65.4 Impact of recent stressors: 60.4 Hostility: 54.4 Marital status: 49.0 History of head injury: 48.2 Psychosocial functioning: 41.2 |  |
| Joo et al., 2022 | Overall suicidal thoughts and behaviors | Multivariate logistic regression | Internal validation was conducted with a testing subset that is 20% of the data | ROC = 0.766, sensitivity = 0.7207, specificity = 0.619 | NA (all prediction results are based on the elastic net model) | NA |
|  |  | Random forest |  | ROC = 0.755, sensitivity = 0.741, specificity = 0.614 | NA (all prediction results are based on the elastic net model) |  |
|  |  | Elastic net regression |  | ROC = 0.766, sensitivity = 0.764, specificity = 0.580, accuracy = 0.672 | Rank (European only): 1. CBCL depressive symptoms 2. CBCL internalizing problems 3. CBCL total problems | Total number of predictors not explicitly stated |
|  | Suicidal ideation | Multivariate logistic regression |  | ROC = 0.736, sensitivity = 0.704, specificity = 0.625 | NA (all prediction results are based on the elastic net model) | NA |
|  |  | Random forest |  | ROC = 0.758, sensitivity = 0.695, specificity = 0.630 | NA (all prediction results are based on the elastic net model) |  |
|  |  | Elastic net regression |  | ROC = 0.759, sensitivity = 0.739, specificity = 0.581, accuracy = 0.660 | Rank (European only): 1. CBCL depressive symptoms 2. CBCL anxious/depressed symptoms 3. CBCL internalizing problems | Total number of predictors not explicitly stated |
|  | Suicide attempt | Multivariate logistic regression |  | ROC = 0.718, sensitivity = 0.494, specificity = 0.485 | NA (all prediction results are based on the elastic net model) | NA |
|  |  | Random forest |  | ROC = 0.946, sensitivity = 0.858, specificity = 0.745 | NA (all prediction results are based on the elastic net model) |  |
|  |  | Elastic net regression |  | ROC = 0.929, sensitivity = 0.858, specificity = 0.818, accuracy = 0.838 | Rank (European only): 1. CBCL total problems 2. We fight a lot in our family 3. CBCL externalizing problems | Total number of predictors not explicitly stated |
| Lozupone et al., 2022 | Suicidal ideation | Random forest | No validation was conducted | NA (AUCs reported for logistic regression models but not for random forest) | Rank: 1. Education (Importance Score = 1.0000) 2. Age (Importance score = 0.9084) 2. Mild cognitive impairment (Importance score = 0.7580) 4. Gender (Importance score = 0.7131) 3. Mini mental state examination (Importance score = 0.6019) | all retained predictors from feature selection |
| Tate et al., 2022 | Suicidal behaviors | Gradient boosted machine | Internal validation was conducted with a testing subset that is 10% of the data and further external validation was conducted with the Netherlands Twin Register | AUC = 0.643, 95% CI = 0.585, 0.702 | Rank: 1. Sex 2. Aggression symptoms 3. Externalizing symptoms | Total number of predictors not explicitly stated |
|  |  | Random forest |  | AUC = 0.617, 95% CI = 0.559, 0.677 | Rank: 1. Aggression symptoms 2. Sex 3. Externalizing symptoms |  |
|  |  | Elastic net |  | AUC = 0.676, 95% CI = 0.620, 0.734 | NA (feature level coefficients were not reported) |  |
|  |  | Neural network |  | AUC = 0.656, 95% CI = 0.601, 0.714 | NA (feature level coefficients were not reported) |  |
| van Velzen et al., 2022 | Child-reported suicidal thoughts or behaviors | Binomial penalised logistic regression | Internal validation was conducted with a testing subset that is defined by data collection sites (7 out of 21 used for testing) | AUROC = 0.715, sensitivity = 0.602, specificity = 0.710, accuracy = 0.656, PPV = 0.674, NPV = 0.640 (from a comparison with clinical control samples and optimal alpha penalization) | Rank (clinical control vs. suicidal thoughts and behavior): 1. Family conflict 2. Prodromal psychosis 3. Impulsivity: negative urgency 4. Impulsivity: lack of planning 5. CBCL depression subscale | all retained predictors from feature selection |
|  | Parent-reported suicidal thoughts or behaviors | Binomial penalised logistic regression |  | AUROC = 0.774, sensitivity = 0.649, specificity = 0.760, accuracy = 0.704, PPV = 0.730, NPV = 0.684 (from a comparison with clinical control samples and optimal alpha penalization) | Rank (clinical control vs. suicidal thoughts and behavior): 1. CBCL anxious/depressive subscale 2. CBCL internalizing 3. CBCL externalizing 4. CBCL depression subscale 5. History of mental health service use 6. History of mental health treatment |  |
| Cho et al., 2021 | Suicide | Random forest | Internal validation was conducted with a testing subset that is 30% of the data | AUC = 0.818, accuracy = 0.832, sensitivity = 0.600, specificity = 0.833, NPV = 0.999, PPV = 0.007 | Rank:  1. Benzodiazepines 2. BMI 3. Age | Total number of predictors not explicitly stated |
| Li et al., 2021 | Recent suicide attempt | Gradient boosted decision trees with extreme gradient boosting | Internal validation was conducted with a testing subset that is 40% of the data | AUC = 0.87, accuracy = 0.87, sensitivity = 0.591, specificity = 0.612 | Rank: 1. Excitement 2. Hostility 3. Hamilton anxiety scale | Total number of predictors not explicitly stated |
|  | Long-dated suicide attempt | Gradient boosted decision trees with extreme gradient boosting |  | AUC = 0.88, accuracy = 0.88, sensitivity = 0.528, specificity = 0.496 | Rank: 1. Hamilton anxiety scale 2. Marriage status 3. Hostility |  |
| Oppenheimer et al., 2021 | Suicidal thoughts or behaviors | LASSO regression | No validation was conducted, but hyperparameter selection was conducted with 10-fold cross-validation | NA (LASSO regression model performance not reported, however follow-up analysis with Poisson regression with selected predictors explained 21.2% of the variance in suicidal thoughts and behaviors) | Age: Lasso coefficient = -0.004, 95% CI = -0.15-0.03 Level of education: Lasso coefficient = -0.01, 95% CI = -0.33, 0.08 HAM-D depression: Lasso coefficient = 0.01, 95% CI = -0.01, 0.05 Kessler Psychological Distress Scale: Lasso coefficient = 0.002, 95% CI = -0.01, 0.05 L. amygdala to sad: Lasso coefficient = 0.08, 95% CI = 0.08, 0.42 | all retained predictors from feature selection out of 114 variables |
| Cho et al., 2020 | Suicide one year follow up | Random forest | Internal validation was conducted with a testing subset that is 30% of the data | AUC = 0.818, accuracy = 0.788, sensitivity = 0.657, specificity = 0.788, PPV = 0.003, NPV = 0.999 | Rank: 1. Moderate exercise 2. Strenuous exericse 3. Alcohol intake | Total number of predictors not explicitly stated |
|  | Suicide overall follow up | Random forest |  | AUC = 0.849, accuracy = 0.754, sensitivity = 0.817, specificity = 0.754, PPV = 0.007, NPV = 0.999 | Rank: 1. Strenuous exercise 2. Alcohol intake 3. Moderate exercise | Total number of predictors not explicitly stated |
| Ge et al., 2020 | Suicidal ideation | Neural network | Internal validation was conducted with a testing subset that is 25% of the data | AUC = 0.743, sensitivity = 0.7068, specificity = 0.6709 | Rank: 1. Free thyroxine 2. Hamilton depression scale 3. Professional skill worker | Total number of predictors not explicitly stated |
| Haines-Delmont et al., 2020 | Suicide risk | K-nearest neighbours | Internal validation was conducted with 10-fold cross-validation | AUC = 0.65, accuracy = 0.68 | NA (feature level coefficients were not reported) | NA |
|  | Suicide risk | Random forest |  | Accuracy = 0.60 | NA (feature level coefficients were not reported) |  |
|  | Suicide risk | Support vector machine |  | Accuracy = 0.57 | NA (feature level coefficients were not reported) |  |
|  | Suicide risk | Logistic regression |  | Accuracy = 0.55-0.59 | NA (feature level coefficients were not reported) |  |

*If the total number of predictors included in the feature space is reported, then we report in this column the top decile of important predictors by rank. Otherwise, the top three predictors are reported. In the case where feature selection algorithms were deployed (such as selecting non-zero coefficients from a penalized regression), we report all retained features as important features.
